# The attractiveness of jobs in the German care sector: results of a factorial survey

**DOI:** 10.1007/s10198-022-01443-z

**Published:** 2022-03-18

**Authors:** Martin Kroczek, Jochen Späth

**Affiliations:** Institute for Applied Economic Research (IAW), Tübingen, Germany

**Keywords:** Nurse, Labour supply, Job choice, Factorial survey, I11, J44, J22

## Abstract

The skilled labour shortage in nursing is an issue not unique to Germany. Unattractive characteristics of nursing jobs are one reason for the low supply in nursing personnel. In our study, we analyse the influence of job characteristics on the attractiveness of nursing jobs. We address this issue via factorial survey analysis, an experimental method particularly suited to assessing personal opinions and less prone to social desirability bias than standard interview methods. Around 1300 (current and former) nurses in a distinct region in Germany were asked to rate a set of synthetic job postings, each of which contained information on 9 systematically varied job characteristics. We find that, first, attractiveness of care jobs is most strongly affected by rather “soft” characteristics such as atmosphere within the team and time for patients. “Hard” factors play a considerably smaller role. Second, one hard factor, contract duration, is estimated to be among the most important job factors, however. This is a remarkable finding given that nursing occupations suffer from severe skill shortages. Third, though wage has a statistically significant influence on attractiveness, enormous wage raises would be needed to yield higher attractiveness gains than the top-rated soft factors, or to compensate for less pleasant job characteristics with respect to those factors. Last, even after controlling for other job characteristics, hospital nursing is still rated as more attractive than geriatric nursing.

## Introduction

For a couple of years, Germany, among other countries, has been suffering from a skilled labour shortage in nursing occupations [1, 2]. Due to their significance for the health sector, the shortage in nursing personnel has received particular attention in the general public as well as in the scientific community. Several countries took up strategies to tackle the problem, such as a push for increased immigration of nurses, changes in nursing training or the implementation of new occupational profiles [3, 4]. Nurses’ labour supply also entered the international research agenda. The literature identifies pecuniary and non-pecuniary job characteristics which can increase nurses’ labour supply [5–10], the time nurses stay with their job or occupation [11–17], nurses’ job satisfaction [18–20], and nurses’ willingness to accept job offers [6, 21–23].

Our paper relates closely to this literature, particularly to the papers analysing nurses’ willingness to accept job offers which typically apply discrete choice experiments (DCEs—see e.g. Doiron et al. [6], Fields et al. [22], Scott et al. [23]; see Soekhai et al. [21] for an overview). Similar to this literature, we analyse potential determinants of the attractiveness of nursing job offers and nurses’ job offer acceptance via a factorial survey experiment, which allows us to receive nurses’ judgements on a randomly assigned set of jobs and estimate the causal effects of potential determinants. This method further allows for insights into the implicit preferences of interviewees and to mitigate a potential social desirability bias of the answers in the survey [24]. Because of its benefits, the factorial survey method has already been applied in the literature on job offer acceptance and job attractiveness [25–27].^1^

To our best knowledge, we are, however, the first to investigate the causal relations of several job characteristics on job offer acceptance and job attractiveness for a cross-section of nurses of all working age groups in a European country in general and for Germany in particular. Results from extant studies for other, even industrialised, countries like Australia or the U.S. can only partly be transferred to the different institutional settings in Europe and Germany. Some previous studies are based on broader groups, like nurses and midwives [23] or nurses and doctors [29], or very specific groups, like highly specialised surgical technologists [30]. Due to the different institutional contexts and analysed groups, previous studies further do not cover all the job characteristics investigated in this paper like contract duration, roster reliability, or institutional setting. Yet, our results show that these factors have a significant influence on nurses’ job offer acceptance.

Our results point to four major findings. First, attractiveness of care jobs is affected most strongly by rather soft characteristics such as atmosphere within the team and time for patients. Hard factors play a considerably smaller role. Second, there exists one very important hard factor: contract duration is estimated to be among the most important job factors, a remarkable finding given that nursing occupations suffer from severe skill shortages. Third, though wages have a statistically significant influence on attractiveness, enormous wage raises would be needed to yield higher attractiveness gains than the top-rated soft factors, or to compensate for less pleasant job characteristics with respect to those factors. Lastly, even after controlling for other job characteristics, hospital nursing is rated as more attractive than geriatric nursing. This finding reinforces the argument for a recent policy change in the German system of vocational training for nurses, where training for geriatric and hospital nurses was unified.

The rest of the paper is organised as follows: in the next section, we present related literature. In Sect. 3, we describe our data and estimation methods. We present our results in Sect. 4. In Sect. 5, we provide concluding remarks and political implications of our results.

## Related literature and hypotheses

How to make nursing jobs more attractive and thereby enlarge the nursing labour supply has aroused research interest in these times of a widespread shortage of nurses. Factors associated with the attractiveness of nursing jobs are analysed through the lens of research on labour supply elasticity, job retention, nurses’ job satisfaction, and job offer acceptance.

Wage raises are one publically advocated measure to cope with the nursing labour shortage [31].^2^ The effects of wage raises on the attractiveness of care jobs are not uncontroversial, however: Shields [9] and Antonazzo et al. [10] provide overviews over research on the wage elasticity of the nurse labour supply. Shields [9] concludes that labour supply is rather unresponsive to wage changes, a conclusion Di Tommaso et al. [8] and Andreassen et al. [5] reach in more recent studies for Norway, too. Antonazzo et al. [10], on the contrary, conclude that the significance of the effect of nurses’ wages on labour supply is rather unclear, as some papers in their review point to significant effects whereas others do not. Differentiating between shift types and occupations in their estimation model and accounting for labour supply decisions on the intensive as well as on the extensive margin, Hanel et al. [7], employing Australian survey data, find a significantly higher wage elasticity of labour supply for nursing degree-holders than former studies without that distinction. There is also evidence that wage level is associated with nurses’ job and occupational retention. Kankaanranta and Rissanen [14] find an association between intent to leave one’s employer and one’s wage as well as the share of income from shift work for a sample of Finnish registered nurses. Frijters et al. [15] and Holmås [16] detect statistically significant effects of the wage level on nurses’ decision to leave the British NHS and the Norwegian public healthcare system, respectively. Their estimated effects differ with respect to their economic significance, though. Doiron et al. [6] evaluate which factors influence job offer acceptance of nurses in Australia by employing a DCE. They find salary to be the most important factor for job selection. This result is driven by the fact that they evaluate a rather large wage rise of over 50%, however. In a study regarding nurses in the US, Fields et al. [22] also find a significant effect of earnings on job choice, but they also find that nurses were willing to give up part of their earnings for other favourable job characteristics. The finding that income is an important but not a dominating job characteristic is in line with findings of further studies on nurses and midwives in Australia, public sector nurses in Malawi, nurses and midwives in Peru, doctors and nurses in China, and nurses in Thailand [23, 29, 34–36]. Wage and wage expectations have similarly been documented as relevant, though not dominating, determinants of whether or not students start vocational training in a nursing occupation [37]. The wage level is also a source of job satisfaction. Lu et al. [19] and Lu et al. [20] provide overviews of the literature on job satisfaction among nurses, identifying remuneration as a source of nurses’ job satisfaction commonly identified in the literature.

It is quite undisputed that non-pecuniary work aspects have a profound influence on the attractiveness of care jobs. Indeed, a vast number of characteristics have been found to be associated with nurses’ intention or decision to start or keep working with an employer, or to stay in the healthcare system or in the nursing occupation. Among those are rather objective, hard factors such as working hours [12, 15–17, 23, 38], shift arrangements [6, 16] and contract duration or time until getting a permanent contract [13, 15, 35, 39]. Furthermore, subjective, soft factors have been found to be associated with the attractiveness of care jobs. Among those are time pressure and quality of care [40], competences and autonomy, and work relationships [22, 23, 40, 41]. Apart from this, a broad range of non-pecuniary factors are also evaluated with regard to their effects on nurses’ job satisfaction. The literature summaries by Lu et al. [20], Lu et al. [19], and Lu et al. [18] also discuss the association between non-pecuniary factors and nurses’ job satisfaction. According to their analyses, the abovementioned factors play a prominent role in job satisfaction literature, too.

The intention or decision to start or keep working in nursing is also associated with individual factors. These include an individual’s family situation, age, experience, tenure [15–17, 40], and ethnic background [17].

Our research interest lies in the determination and quantification of the effects of job characteristics on job attractiveness and job offer acceptance. A vast set of factors has been evaluated in the literature, which can be divided into three major categories: pecuniary job factors, particularly wage; objective (or hard) non-pecuniary job factors such as working hours; and subjective (or soft) non-pecuniary job factors, such as autonomy. Objectively measurable factors like wage or working hours have produced a greater extent of quantitative literature analysing their effects than is the case for rather subjective factors like autonomy. However, although a majority of the cited literature points to a positive effect on the attractiveness of nursing jobs (e.g. labour supply, job choice, job satisfaction and job retention), the effect of wage is not undisputed, be it in terms of its mere existence or in terms of its size. Differences in the estimated sizes of the wage effect seem to evolve along different outcomes evaluated (e.g. job retention vs. job acceptance), methodological approaches and region. Also, extant results are not easily transferable to the institutional settings in European countries, such as Germany. In accordance with the literature, we hypothesise that pecuniary as well as objective and subjective non-pecuniary factors significantly influence job attractiveness and job offer acceptance–though it is a priori unclear how large the absolute or relative effects of factors from the three domains are. Employing the factorial survey method, we test the significance of factors from those domains for job attractiveness and quantify their absolute and relative influence using a broad sample of nurses in a European country. To our best knowledge, no such experimental analysis of nurses’ job preferences for a cross-section of nurses of all working age groups in a European country exists.

How individual factors influence the interest to work in specific nursing jobs is not a concern of this paper, as we study the attractiveness of care jobs rather than the full set of determinants of labour supply in care.

## Institutional background

Some peculiarities regarding the provision of care services in Germany have to be considered when analysing the attractiveness of care jobs in the German context. Care personnel in Germany have long been divided into different groups according to their main areas of action as well as their levels of occupational training. Whereas geriatric nurses mainly work with elderly people, hospital nurses mainly work in care for the sick. Further, geriatric and hospital nurses may have the occupational education to work as a registered geriatric or hospital nurse or as a geriatric or hospital nursing assistant according to their vocational education. This separation along areas of care and level of occupational education is grounded in the history and education system of care in Germany [42]. The different areas of activity could be associated with different levels of attractiveness, and geriatric nursing has been found to be viewed as less attractive than hospital nursing.^3^ This differentiation should be considered, as we surveyed German nurses in this study.

It is not only occupations that are differentiated between care for the elderly and care for the sick in Germany. Reimbursement rules also differ. Nursing services in healthcare are mainly paid for by health insurances, which cover the full amount of the respective costs. Geriatric nursing services are paid for by long-term care insurance, which only covers a share of long-term care expenditures; the rest of the long-term care expense has to be borne by the care recipient [42]. Also, the services provided and the amounts institutions can charge for services in health as well as geriatric care are strongly regulated and differ between outpatient geriatric care, outpatient health services, hospital care services and inpatient geriatric services [45].

These regulations limit organisations’ freedom regarding the provision of the respective services and set limits to how care work is organised—limits we had to consider when we set up the hypothetical job offers. The reimbursement rules in the provision of health and geriatric services further limit the range for nurses’ wages, bounding nurses’ wages from above. On the other hand, nurses’ wages are also bounded from below due to a specific minimum wage for nurses [46]. Within these boundaries, wages for nurses in hospitals are further regulated, as they are mostly employed under the rules of collective agreements. In geriatric nursing, collective agreements are less common [42]. The institutional situation of bounded and partly collectively regulated wages leads to a situation where nurses’ wages are not as flexible as in other sectors. However, nurses’ wages still exhibit significant variation, as Bogai et al. [47] show. In our context, this allows for (synthetic) job offers that contain a significant range of wages without offering too unrealistic wage rates. Still, as low as employers’ leeway regarding the establishment of favourable working conditions may appear in general, there are still some adjustment screws.

## Data

### Survey method

To assess the question of which factors drive job attractiveness and job offer acceptance, we ran a standardised survey among current and former care workers. As we exclusively sampled persons who work or have in the past worked in care, we focus on what attracts the core nursing labour potential (back) to nursing jobs. To study how new segments of the population could be attracted into care professions, individuals who never worked in nursing would have to be interviewed, too. In setting up the survey, the following considerations were taken into account: (1) care workers do not need to be explicitly aware of each and every of their own preferences, which ultimately influence their perception of the attractiveness of a given job. (2) Even if they were, several aspects could be highly correlated and thus difficult to disentangle if respondents were asked directly about their influences on job offer acceptance. (3) Among other factors, our survey involves issues that are likely to suffer from social desirability bias, such as the hourly wage or whether care professionals prefer to have much excess time for patients.

To account for these aspects, the survey consisted of two parts. One part of the survey was traditionally item-based and served the purpose to query control variables. The main part of the survey was designed as a factorial survey where care workers were presented with vignettes describing fictional advertisements of care jobs.^4^

A factorial survey differs from a traditional item-based survey insofar as several parameters relevant to the research question are enquired in a coherent unit of meaning at the same time, instead of asking several separate questions. Thus, respondents to a factorial survey always evaluate an overall set of variables that are interrelated and can influence each other. When combining multiple variables with multiple characteristics, many different vignette texts are possible. By presenting several systematically varied constellations, the influence of the individual dimensions can be separated in the analyses.

### Survey design

As part of the factorial survey, the fictional job advertisement (see Table 1) was presented to care workers in form of a series of vignettes in text form. The vignettes follow a 2^7^3^1^8^1^ experimental design, which generates a systematic variation of the individual text modules. To measure the influence of pecuniary job characteristics, the vignettes contain wage as one dimension. To access the influence of soft non-pecuniary characteristics, work autonomy, time for patients, atmosphere within the team and roster reliability enter the vignettes. We address the influence of hard non-pecuniary job characteristics via the type of care activity (hospital or geriatric nursing), the kind of care institution (in- or outpatient care), working hours and contract duration. We selected the specific dimensions for the respective domains in a multistage process which included an exploration of the relevant literature, expert interviews and pretests to identify the most prominent or most relevant dimensions. The dimensions of care activity and care institution are not prominent in the international literature; differentiation along those characteristics is important in the German context, however. Reading from top to bottom, the right column of Table 1 lists all conceivable vignette constellations.

**Table 1:**
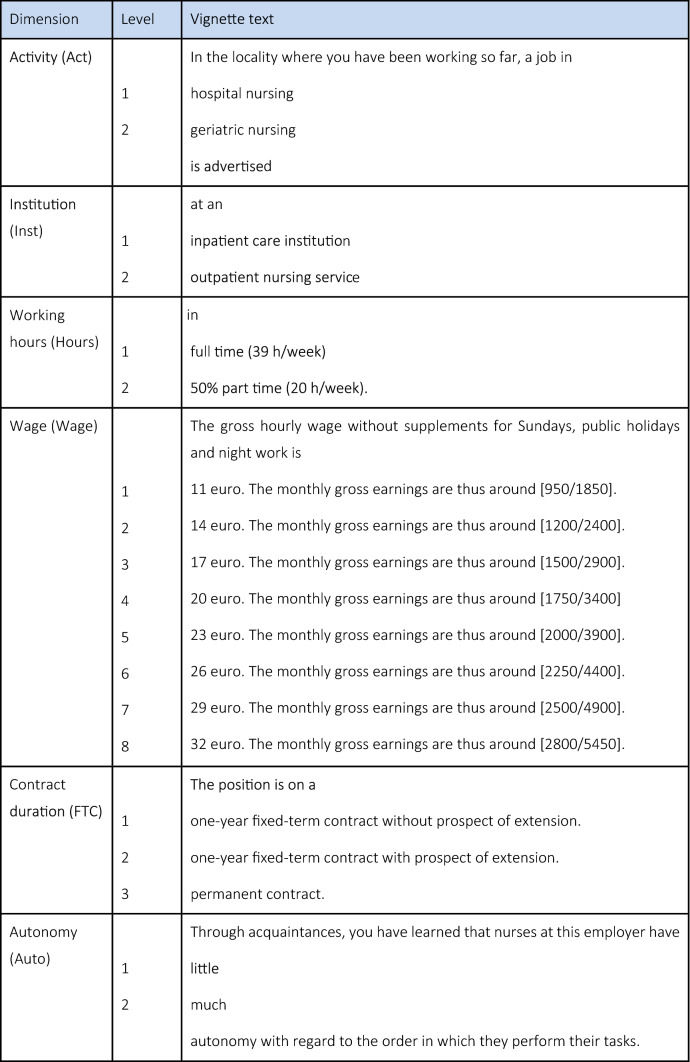

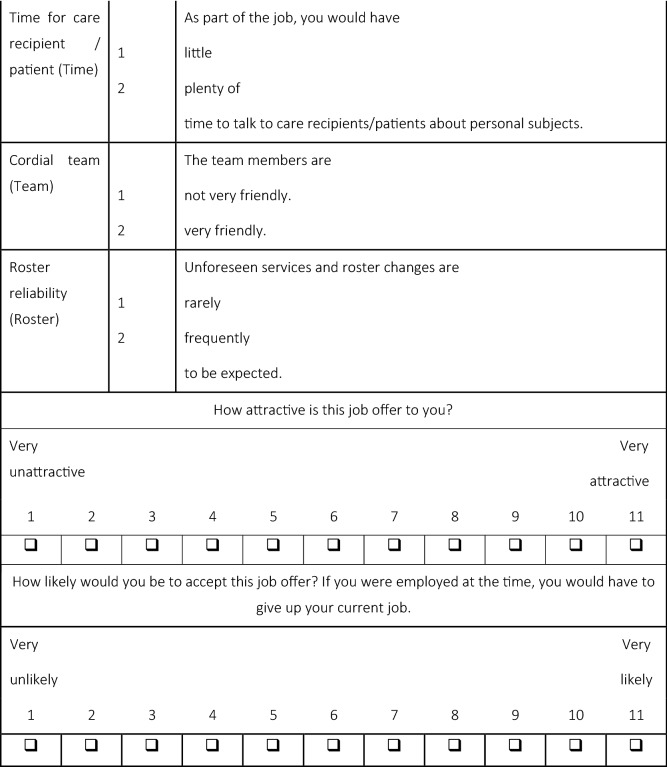
Vignette dimensions and levels

Source Factorial survey of (former) care workers, own representation

To reduce the survey load, a fractionalised^5^ sample of 200 vignettes was drawn from all possible combinations of vignette dimensions and distributed to the various questionnaires by fractionalised blocking, so that each respondent received 10 different vignettes. The questionnaires were randomly assigned to the respondents to ensure the vignette variables were independent from respondent characteristics. The survey uses a Resolution V design with a D-efficiency^6^ of 95. The results thus have a high internal validity. In concrete terms, all the main effects, along with all two-way interactions of the vignette dimensions, can be estimated in isolation from each other. Analysing our final sample, we do not find two single dimensions with a correlation coefficient larger than 0.1.

The care workers evaluated (a) how attractive the presented job offer is to them and (b) how likely they would accept that job offer, under the condition that they would have to give up their current job if they had one.

To estimate the causal effects of the vignette dimensions on the attractiveness of the fictitious jobs, we first relied on the experimental design, which allowed us to separate the effects of the single dimensions. Second, to abstract from the fact that the evaluation of the vignettes might be affected by other unknown confounding variables, the vignette text was preceded by a *vignette intro.* In the intro, essential confounders, which possibly affect job attractiveness, but are not under control of the employer or not in the focus of this study (e.g. commuting distance from home), were “controlled” by being (fictitiously) held at a level that remained constant over the vignettes.

The survey underwent several rounds of cognitive pre-testing with experts and nurses.

### Caveats and limitations

Though factorial surveys offer several advantages, some issues need to be addressed in designing such a survey. Generally, the vignettes, together with the vignette intro, need to contain all information relevant to rate the vignettes. Also, factorial surveys evaluate hypothetical situations, not actual situations. Other than this, some more specific issues need to be considered.

As factorial surveys are more complex than standard surveys, particular effort is needed in order not to overburden respondents with the survey setup. Specifically, the number of the vignettes presented to every respondent, the number of dimensions every vignette contains as well as the levels per vignette dimension need to be considered. Although a larger number of dimensions would enable us to study more job characteristics, vignettes with too many dimensions can become too complex, overburden respondents and lead to inconsistent judgments [50]. Also, if respondents are presented with too many vignettes, they have been found to show fatigue effects or employ specific rating heuristics [50, 51]. We, therefore, follow the methodological literature, which suggests (depending on the concrete setup) a maximum number of nine vignette dimensions and ten vignettes per respondent [24]. The subjects in our cognitive pretests did not report being overburdened by the number of vignettes or the processing of the vignette text itself. Regarding the number of levels per dimension, we must consider several factors. Although a finer scaling of the dimensions enables a finer differentiated effect estimation, this would also mean that a larger set of vignettes would have to be evaluated. In order to get efficient results, a limitation on a subset of all possible scale values is needed.^7^ Seven of the dimensions in our experimental setup, therefore, contain two levels, only. This could become problematic if we chose too extreme levels for the dimensions with only two levels. We considered the scaling of all dimensions during the cognitive pretests. The pretests did not point to a too extreme understanding of the alternatives. And even if one were to believe, the two levels were extreme, they can be seen as an upper bound to the effect arising from more fine-grained levels.

As we pointed out, all vignette studies are limited in the number of dimensions included. On the other hand, the vignettes, together with the vignette intro, need to contain all information relevant to rate the vignettes. Specifically, the results of an experiment could be biased if individuals associate single levels of included dimensions with characteristics which are not included. In setting up the vignettes for our research question, we chose single characteristics for the three domains of pecuniary, objective non-pecuniary and subjective non-pecuniary job factors from a wide range of potential characteristics in a multistage process. We identified the relevant dimensions from national and international literature and expert interviews and verified the choice of the final set in two rounds of cognitive pretests. We incorporated relevant characteristics which did not become part of the vignettes in the vignette intro. The results of our cognitive pretests make us confident that we did not miss out on information which would influence the results of our study.

We also must consider the extent of the response scale. If too extensive response scales are used, individuals may bunch at round numbers or neutral values [51]. We, therefore, employ a 11-point scale, which has been found to work well in the literature [24].

### Sampling procedure and sample characteristics

The survey was addressed to current and former nurses who work or who have worked in the regional planning area (RPA) of Heilbronn-Franken, Germany. RPAs are constructed for the purpose of carrying out nationwide comparisons and assessing large-scale regional tendencies and explicitly rely on the concept of an economic centre surrounded by a more rural periphery. The precise RPA of Heilbronn-Franken was chosen as a region which is comparable to the regions surrounding it with respect to socio-demographic factors, and as it is not adjacent to other national states (Austria, France, Switzerland) because proximity to a national border would bring about specific peculiarities that could affect the intended analyses.

Our gross sample was drawn from the Integrated Employment Biographies (IEB) from the Federal Employment Agency (BA)^8^ and consisted of 8116 individuals who worked as professional caregivers in the named RPA at the time of the sampling or who had worked as professional caregivers in the RPA during the 5 years prior to sampling. The sample was stratified by employment status (currently vs. formerly working as a care professional), (former) profession (nurses vs. geriatric nurses), and the kind of institution the respondents (formerly) worked in (inpatient vs. outpatient care).^9^ Within these strata, interviewees were drawn at random.

The IEB contain process data at the person level from the procedures of the BA and cover almost all individuals who have been in paid employment at some point [53].

Data collection was carried out in the form of paper and pencil interviews (PAPIs) in November and December 2018. To generate high response rates, the participants also received a letter of recommendation from the German Nurses Association Southwest (DBfK). Participation was further incentivised via vouchers for a big internet retailer given to the first 800 respondents to mail back their questionnaire. We received a total of 1607 filled-out questionnaires (around a 20% response rate). After data cleansing, we were left with 1313 completed interviews.^10^ More than 1000 of those correspond to active nurses. Our net sample is comparable to the gross sample in terms of the sex and age of respondents. However, German, currently active registered nurses (with completed vocational training) working in a stationary care setting were overrepresented.^11^

Table 2 gives an overview of the basic sample distribution across care professions and care status. Nearly half of all respondents in our sample belong to the nursing care sector, about one-third belong to elder care and about one-fifth claim to work in both nursing care and elder care at the same time. Nearly four-fifths of the sample consist of current nurses, while the remaining fifth is constituted by former nurses. The former nurses in our sample belong to nursing care more frequently than the current nurses do, and in elder care, there are fewer former nurses than across all care sectors.

**Table 2:** Care worker sample by care sector and nurses’ care status (current vs. former nurses). Source Care worker survey, own calculations

		Current nurses	Former nurses	Total
Nursing care	*n*	461	153	614
	Row %	75%	25%	100%
	Column %	44%	56%	47%
Elder care	*n*	355	43	398
	Row %	89%	11%	100%
	Column %	34%	16%	30%
Both at the same time	*n*	224	77	301
	Row %	74%	26%	100%
	Column %	22%	28%	23%
Total	*n*	1040	273	1313
	Row %	79%	21%	100%
	Column %	100%	100%	100%

Beyond the information presented in Table 2, about two-thirds of the current nurses work in inpatient care, yet with considerable differences between care sectors. Although nurses working in inpatient care account for the majority of respondents from nursing care and elder care (83% and 72%, respectively), only every tenth respondent working in both nursing care and elder care at the same time (and in the same job) belongs to inpatient care. About nine out of ten respondents are female and German. Average age is 45 years. 85% of the respondents have at least 5 years of experience in direct care. All respondents have completed vocational training as a nurse. More than half of the respondents have a secondary school diploma (German *Realschulabschluss*).

## Methods

We estimate the relationship

1 $${y}_{ij}=\mu +{{\varvec{x}}}_{j}^{\boldsymbol{^{\prime}}}{\varvec{\beta}}+{\alpha }_{i}+{\epsilon }_{ij},$$

where ${y}_{ij}$ denotes the attractiveness or job offer acceptance evaluation of job offer $j$ for person $i$, $\mu$ is a constant, ${\varvec{x}}$ is a vector of observable characteristics of the job offer, ${\varvec{\beta}}$ is a vector of parameters measuring the influence of the observable characteristics on the attractiveness evaluation, $\alpha$ is an unobserved individual effect at the person level, and $\epsilon$ is an individual and job offer-specific error term.

When set up effectively, the factorial survey design virtually rules out endogeneity issues related to the vignette dimensions, as correlation between them is eliminated as much as possible and surveys are assigned to each respondent at random. The job characteristics are therefore uncorrelated with the individual and job-specific error term $\epsilon$ (strict exogeneity assumption) and the unobserved individual effect $\alpha$ by design (random effects assumption) [24, 54]. We, therefore, estimate random effects (RE) models, which yield more efficient estimates of the effects of the job characteristics than the standard OLS estimator [24, 54].^12^

An instructive way to grasp the size of the effects of the different characteristics on job offer acceptance and job attractiveness is how much extra wage an individual would have to be compensated with for a less pleasant job characteristic, meaning by how much pay would have to rise for a job offer to be ranked as attractive as another, otherwise equal job offer, where one characteristic $k$ is more positive (${x}_{k}$ changes from 0 to 1). In this case, the overall change in job attractiveness would be zero, and hence

2 $${\beta }_{w}\mathrm{log}\left(wage+\Delta wage\right)={\beta }_{w}\mathrm{log}\left(wage\right)+{\beta }_{k},$$

where $\Delta wage$ is the change in wage retained as compensation for the less pleasant working conditions. Re-transforming wage to the original scale and rearranging this equation, we get

3 $$\mathrm{\Delta wage \: in \: \%}= \left[\mathrm{exp}\left(\frac{{\upbeta }_{\mathrm{k}}}{{\upbeta }_{\mathrm{w}}}\right)-1\right]*100,$$

which yields the compensation needed to keep job attractiveness constant when ${x}_{k}$ changes from 1 to 0 (Auspurg and Hinz [24] propose a comparable approach). As an example, this compensation measure answers the following question: How much more does an employer have to pay for a job on a fixed-term contract compared with a job on a permanent contract in order for two otherwise comparable job offers to be similarly attractive?

## Results

Table 3 shows the RE estimates of the effects of the vignette dimensions on job attractiveness (column 1) and the willingness to accept a job offer (column 2). The dependent variables in our models are measured on an 11-point scale. Therefore, the absolute size of the coefficient of a job characteristic shows, by how much the rating of a job offer changes, if the characteristic changes by one unit (i.e. from a less favourable to a more favourable manifestation or from hospital nursing to geriatric nursing and from inpatient care to outpatient care, respectively). Wages are an exception and

**Table 3:** Main effects of job characteristics on job attractiveness and job acceptance, RE estimation results. Source Care worker survey, own calculations

	(1)	(2)
	Attractiveness	Acceptance
	Linear RE	Linear RE
Hospital nursing	0.144*** (0.038)	0.118*** (0.035)
Outpatient nursing service	0.066 (0.043)	0.038 (0.041)
Full time (39 h/week)	0.463*** (0.045)	0.389*** (0.043)
ln (hourly wage)	3.503*** (0.074)	2.679*** (0.073)
1-year FTC with prospect of extension	0.665*** (0.048)	0.585*** (0.045)
Permanent contract	0.914*** (0.050)	0.832*** (0.046)
Much autonomy of how to work	0.517*** (0.041)	0.389*** (0.039)
Plenty of time for care recipients/patients	1.173*** (0.042)	0.881*** (0.040)
Very friendly team	1.339*** (0.042)	1.015*** (0.041)
Reliable roster	0.729*** (0.041)	0.584*** (0.038)
Constant	− 9.498*** (0.220)	− 7.336*** (0.219)
Observations	12,758	12,700
Adjusted/overall R2	0.297	0.211
Rho	0.264	0.294

*/**/*** = significant at the 10/5/1% level. Standard errors clustered at the care worker level

Vignette dimensions and their manifestations (reference categories are underlined)

Activity: *Hospital nursing* and *geriatric nursing.* Institution: *Inpatient care* and *outpatient nursing service*. Working Hours: *Full time* and *50% part time*. Wage: *eight wage levels*. Contract duration: *One-year fixed-term contract without prospect of extension*, *one-year fixed-term contract with prospect of extension*, *permanent contract*. Autonomy: *Little autonomy* and *much autonomy*. Time for patient: *Little time for patient* and *plenty of time for patient*. Team: *Not very friendly* and *very friendly*. Roster reliability: *Rare unforeseen services and roster changes* and *frequent unforeseen services and roster changes*

discussed separately.

First, we note that all coefficients have the expected positive sign and almost all characteristics have a statistically significant effect on job attractiveness and job acceptance throughout the models. Furthermore, the results differ only slightly between the models. The order of the effect sizes is similar with regard to job attractiveness and willingness to accept a job offer (see below). In absolute sizes, the estimated effects on job acceptance (including the constant) are considerably smaller, though, which could be due to the more serious consequences implied by the question regarding job acceptance relative to the attractiveness rating.

The sizes of the estimated coefficients differ considerably. When we arrange the different factors by effect size from largest to smallest, we get the following order: 1. Team, 2. Time for patients, 3. Contract duration, 4. Roster reliability, 5. Autonomy, 6. Volume of work, 7. Care sector, and 8. Care institution. The effect of the wage variable is not part of this enumeration due to its continuous nature and will be discussed separately. Notably, with the exception of contract duration, we estimate the largest effects for rather soft factors of work atmosphere and organization of work. Working with a very cordial team as opposed to a less cordial team increases attractiveness and acceptance ratings by more than one point on the 11-point scales. An offer for a job in which staff has more time for patients is also rated higher by around one scale point. Working on a reliable roster gains job offers around 0.7 scale points on the attractiveness scale, and just below 0.6 points on the acceptance scale. More autonomy leads to a 0.5 scale-point gain on the attractiveness scale and just below a 0.4-point gain on the acceptance scale. Hard job factors such as the volume of work and employment in the care sector have a lower influence on job attractiveness. One hard job factor that has a large impact on job attractiveness is contract duration. The rating difference between a job offer with a one-year fixed-term contract without prospect of extension and a permanent contract amounts to around 0.9 and just over 0.8 points on the attractiveness and the acceptance scale, respectively. Although this finding is in line with results from other studies [13, 15, 35, 39], it seems puzzling in times of skilled labour shortage in nursing. It seems that (former) nurses are either not aware of the fact that skill shortage gives them advantages in the labour market or have another reason to value long contracts especially highly; this could be because they are particularly risk averse, feel less valued if they are offered fixed contracts, or simply do not like to change their employer because they want to work with the same team for as long as possible or fear the need to change location if they search for a new job. On the other hand, with between 20 and 30% of contracts, an unexpectedly high share of nurses have been working under fixed-term contracts in Germany in recent years. Although the numbers differ between sources, they are non-negligible throughout them, whether from surveys [55] or from personal calculations based on administrative data on German employment histories (SIAB).^13^ The high shares are surprising, as employers should have an incentive to tie nurses to them as long as possible and offer attractive working conditions due to skill shortage. Furthermore, we see from other publications and our own calculations based on the SIAB that the share of fixed-term contracts in the care occupations has been significantly higher than the average share over all other occupations [55, 57] and other occupations subject to skilled labour shortage in recent years. The latter in particular is unexpected as the demand for care services can almost surely be considered to rise, for instance, due to demographic change [58, 59]. A specific aspect of nursing work in Germany is the division of nursing occupations into hospital nurses, mainly caring for the sick, and geriatric nurses, mainly caring for the elderly [42]. We find that, even after controlling for the other job characteristics in our model, geriatric nursing is still considered significantly less attractive than hospital nursing, which is in line with results from previous (survey) studies in Germany [43, 44]. As we control for several characteristics which usually separate geriatric from hospital nursing jobs (e.g. lower wage in geriatric nursing [47]), our hypothesis is that we measure the overall worse image of geriatric nursing in this way.

Although it is difficult to compare our results with earlier research quantitatively due to significant differences in the scientific approaches and the methods applied, qualitatively, our results for the importance of non-pecuniary job characteristics are in line with earlier work on nurses’ intention or decision to start or keep working with an employer, or to stay in the healthcare system or in the nursing occupation, which found non-pecuniary factors to have significant effects on the named domains of nurses’ labour supply [6, 12, 13, 15–17, 22, 23, 35, 38–40].

Although the ordering of the effects is the same in the job attractiveness model as in the job acceptance model, the effect sizes differ less strongly in the job acceptance model. When confronted with the more serious decision about actually quitting one’s job for a new job offer, objective factors seem to gain relative importance in comparison to a ranking in attractiveness.

An intuitive way to grasp the quantitative relevance of the different job characteristics is the wage change percentage an individual would have to receive to be compensated for a less attractive manifestation of a specific job characteristic. Table 4 gives the respective calculations. The ordering of the amounts of compensation is naturally the same as the ordering of the effect sizes. However, in this way, the relevance can be quantified in a monetary way. Looking at job attractiveness, wage would have to rise by 47% to compensate for a less cordial team, 40% to compensate for less time with patients, 30% to compensate for a fixed-term contract without a chance of prolongation instead of a permanent contract and 23% to compensate for a less reliable roster. The considerable size of these wage compensations already implies that wage itself—though statistically significant—may not play the largest role for care workers’ perception of jobs.

**Table 4:** Compensation for worse working conditions. Source Care worker survey, own calculations

	(1)	(2)
	Attractiveness	Acceptance
	Linear RE	Linear RE
Hospital nursing	0.042	0.045
Outpatient nursing service	0.019	0.014
Full time (39 h/week)	0.141	0.156
1-year FTC with prospect of extension	0.209	0.244
Permanent contract	0.298	0.364
Much autonomy of how to work	0.159	0.156
Plenty of time for care recipients/patients	0.398	0.389
Very friendly team	0.466	0.461
Reliable roster	0.232	0.243

Vignette dimensions and their manifestations (reference categories are underlined)

Activity: *Hospital nursing* and *geriatric nursing.* Institution: *Inpatient care* and *outpatient nursing service*. Working Hours: *Full time* and *50% part time*. Wage: *eight wage levels*. Contract duration: *One-year fixed-term contract without prospect of extension*, *one-year fixed-term contract with prospect of extension*, *permanent contract*. Autonomy: *Little autonomy* and *much autonomy*. Time for patient: *Little time for patient* and *plenty of time for patient*. Team: *Not very friendly* and *very friendly*. Roster reliability: *Rare unforeseen services and roster changes* and *frequent unforeseen services and roster changes*

In addition, as shown in Table 3, a wage increase by 1% increases job attractiveness by about 0.035 points. To approximate the effect of a realistically possible wage change, we adhered to the latest rise in the minimum wage for qualified nursing assistants in Western Germany: over the course of 2020 and 2021, minimum wages for qualified nursing assistants^14^ in Western Germany increased from 11.35 to 12.50 euros, which corresponds to a 10-percent rise in wages.^15^ For those hospital nurses working under a public service collective agreement, a wage increase of 8% has been gained with the 2019 collective agreement. Thus, wage raises of this size would by far have a smaller impact on job attractiveness and job offer acceptance than most of the other job characteristics we evaluated.^16^ Our results are, therefore, in line with the large part of the literature that estimates the effects of wage changes on labour supply and retention of nurses that are so small in size that substantial wage increases would be required to yield economically significant effects on the supply of nurses [5, 8, 9, 15]. Our results with regard to wages are further in line with results of DCEs on nurses’ job preferences which found earnings to have a significant but not dominating effect on job choice [22, 23, 29, 34–36].^17^

We also evaluated the interaction effects between vignette dimensions, as well as between vignette dimensions and respondents’ current work situation. Table 5 provides an overview of the results regarding the former interaction, and statistically significant effects are marked by grey bars. Evaluating the interactions, we observed three interesting points. First, we found hardly any interaction effect between activity or institution and other vignette dimensions. This means that the same factors are relevant for nursing care and elder care as well as for inpatient care and outpatient care jobs, and therefore, the same policies could increase the attractiveness of jobs among the different institutions and activities. Second, wherever we found significant interaction effects between dimensions, they were mostly positive. This means that changes that increase attractiveness amplify each other. Third, we found the largest interaction effects for interactions with the factors that exhibited the largest main effects. Therefore, it could be a rewarding strategy for employers and policymakers to improve simultaneously on more than one of the dimensions with the largest effects. Due to the interaction effects between the evaluated job characteristics, it becomes increasingly costly for an employer to compensate (possible) employees for less pleasant working conditions with respect to more than one job characteristic. Put another way, with an increasing number of unpleasant job characteristics, employers’ wage offers must increase over-proportional.

**Table 5:**
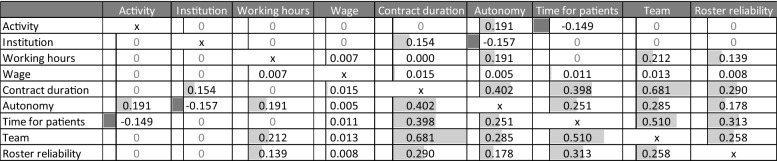
Two-way interactions of vignette dimensions. Source Care worker survey, own calculations

Standard errors clustered at the care worker level. Dependent variable: job offer acceptance. Significant positive interaction effects are marked by light grey bars, and significant negative effects are marked by darker grey bars. A value of “0” indicates that there is no significant interaction effect between two variables

Vignette dimensions and their manifestations (reference categories are underlined)

Activity: *Hospital nursing* and *geriatric nursing*. Institution: *Inpatient care* and *outpatient nursing service*. Working Hours: *Full time* and *50% part time*. Wage: *eight wage levels*. Contract duration: *1-year fixed-term contract without prospect of extension, permanent contract*. Autonomy: *Little autonomy* and *much autonomy*. Time for patient: *Little t﻿ime for patient* and *plenty of time for patient*. Team: *Not very friendly* and *very friendly*. Roster reliability: *Rare unforeseen services and roster changes* and *frequent unforeseen services and roster changes*

Table 6 shows the interaction effects between vignette dimensions and respondents’ current work situation. The results indicate that all but geriatric nurses and nurses working in outpatient care prefer healthcare jobs to jobs in geriatric care. Regarding in- and outpatient care, we found that nurses prefer jobs in the same kind of institution they are or have been employed in—that is, nurses in inpatient care prefer jobs in inpatient care, and those in outpatient care prefer jobs in outpatient care. Another difference can be found with respect to full-time versus part-time jobs. All but former nurses show a preference for full-time jobs. Regarding wages and team spirit, we found effects of the same direction for all groups of nurses, although the effects differ in size. Regarding the other job characteristics (contract duration, autonomy, time for patients and reliable rosters), we found no differences with respect to the different groups of nurses. The effects are, therefore, quite comparable across the different groups of interviewees. However, there may exist variation in the ratings on the personal level over further individual characteristics. A subject of separate, ongoing research is to what extent groups with differing individual characteristics value specific job characteristics differently.

**Table 6:** Cross-level interactions. Source Care worker survey, own calculations

Effect	Hospital nurses	Geriatric nurses	Current nurses	Former nurses	Nurses in inpatient care	Nurses in outpatient care
Activity	+	0	+	+ +	+	0
Institution	0	0	0	0	−	+
Contract duration	+	+	+	0	+ +	+
Wage	+	+ +	+ +	+	+ +	+
Team	+	+ +	+	+	+	+ +

Interactions between respondent characteristics (horizontally) and vignette dimensions (vertically) are expressed

0: no interaction effect, + : positive interaction effect, +  +  positive interaction effect, which is significantly larger than the effect for the other respective category, **−**: negative interaction effect

Standard errors are clustered at the care worker level. Dependent variable: job offer acceptance

Vignette dimensions and their manifestations (reference categories are underlined)

Activity: *Hospital nursing* and *geriatric nursing*. Institution: *Inpatient care* and *outpatient nursing service*. Contract duration: *one-year fixed-term contract without prospect of extension*, *permanent contract*. Wage: *eight wage levels*. Team: *Not very friendly* and *very friendly*

## Summary and conclusions

Many countries, among them Germany, are suffering from a shortage of nursing personnel. How to tackle this shortage has become a topic of major interest for politics and social sciences. In this paper, we explicitly analyse the influence of job characteristics on the attractiveness of nursing jobs. Using factorial survey methods on a self-conducted survey of (former) nurses, we identified important characteristics for job attractiveness, as well as job offer acceptance, and quantified their influence. Our study is related to the research on nurses’ labour supply, nurses’ job and occupation retention and nurses’ job choices. However, to our best knowledge, we are the first to investigate the causal relations of several job characteristics on job offer acceptance and job attractiveness regarding a cross-section of nurses of all working age groups in a European country and for Germany in particular.

We identified four major findings from our results. The first is that the attractiveness of care jobs is strongly affected by non-pecuniary job characteristics—a finding earlier studies reached as well [6, 12, 13, 15–17, 38, 40]. We further found, however, that attractiveness is affected most strongly by rather soft job characteristics, such as atmosphere within the team and time for patients. Rather hard factors play a considerably smaller role.

The second major finding is that there exists one hard job factor that is very important: contract duration is estimated to be among the most important job factors for job attractiveness and job offer acceptance; although this is in line with results from previous studies [13, 15, 35, 39], it is a remarkable finding regarding jobs in occupations exhibiting severe skill shortage. For one thing, this is remarkable, because nurses should easily find a new job once losing their present one. The disapproval of fixed-term contracts may, therefore, be a display of nurses’ strong preferences for safe employment contracts and against new work environments. This is further remarkable in so far as fixed-term contracts still exist (20–30% of nurses are working under a fixed-term contract) in times of skill shortage and the share of fixed-term contracts is even higher than the average share over all occupations and other occupations with skilled labour shortage [55, 57; own calculations based on SIAB]. Rather, employers should have an incentive to offer permanent contracts because it will be difficult for them to hire new nurses if they leave after the end of the fixed term due to nursing skill shortage and because permanent positions would be an effective way to increase attractiveness. Why employers offer fixed-term contracts in times of skill shortage in a part of the economy where demand is almost sure to rise [58, 59] is open to future research. First possible explanations may lie in the reaction of firms to heterogeneous worker preferences [60], an extensive screening phase of firms to guarantee quality standards or a fixed time horizon in the case of parental leave replacement.

The third major finding deals with wages. Although the wage has a statistically significant influence on attractiveness, enormous wage increases would be required to yield higher attractiveness gains than the top-rated soft factors or to compensate for less pleasant job characteristics with respect to those factors. As a consequence, monetary compensation for unpleasant working conditions will be costly. To compensate for unpleasant working conditions with respect to the most relevant job characteristics (e.g. team, time with care recipients/patients, contract duration) wage raises between 20 and 47% would be necessary. This is far from what policymakers and employers were willing to offer in minimum wage raises or in collective agreements so far. If employers and policymakers want to significantly increase the attractiveness of nursing jobs and are not willing to provide substantial wage raises, changes in other job characteristics will be necessary. Especially rewarding strategies will be those providing improvement on more than one of the relevant job characteristics.

The last major point deals with a German peculiarity: the separation between geriatric and hospital nursing occupations. Our results show that, even after controlling for other job characteristics, hospital nursing is still rated more attractive than geriatric nursing. This is, for one thing, in line with what we assumed from previous literature [43, 44]. This finding, for another thing, reinforces the argument for a recent policy change in the German system of vocational training for nurses, where training for geriatric and hospital nurses was unified [61]. In light of our findings, one could argue that the change to a more generalist training for nurses could at least increase the attractiveness of training to become a geriatric nurse.

## Data Availability

Data acquisition was conducted based on data from the German Federal Employment Agency. Data usage was based on a contract with the German Federal Employment Agency. The data are not sharable due to data protection.

## Vignette intro

“Jobs differ in many ways. The following part of the survey contains descriptions of some fictitious job offers in nursing and care for the elderly. All vacancies are fictitious but could happen in reality. We selected all features of the offered positions at random.

At the end of each job description, we would like to hear from you about how attractive the job offered is to you and how likely you would be to accept it if it were offered to you. To do this, we ask you to place your cross on the attached response scales according to your assessment. You can answer the job descriptions in any order. If necessary, you can correct your answers at any time during the survey.

Please note the following:

1. All offered positions are activities in direct nursing care.
2. The prerequisite for the start of all positions offered is a completed training as a specialist in health and nursing, health and child care or geriatric care.
3. All jobs are in the same place as your previous job.
4. There are enough quality childcare facilities near all jobs.
5. At all workplaces, there is a normal shift operation with industry-standard extent of night and weekend services as well as on-call duty.
6. All employers have an average number of employees or beds.
7. All employers are highly regarded in nursing circles.”

Source Care worker survey.

## Footnotes

1 Abraham et al. [27], Auspurg and Gundert [26], and Bähr and Abraham [25] all employ wave V of the Labour Market and Social Security Panel (PASS [28]).

2 Whether and how wage raises can be achieved and which role individual characteristics and institutional settings play in this context lies beyond the scope of this paper. See e.g. Ariste and Béjaoui [32] or Hirsch and Schumacher [33] for respective research.

3 Compared to geriatric nursing, hospital nursing not only has a better image among pupils and their parents, but pupils’ motivation to take up geriatric nursing is also lower than their motivation to take up hospital nursing [43, 44].

4 A detailed description of the methodology of the vignette survey would take up too much space here. A great introduction can be found in Auspurg and Hinz [24].

5 This means a sample with maximum information in the sense of the orthogonality of the vignette dimensions is drawn (“efficient design”). The higher information density of fractionalised samples as opposed to random samples is evidenced by research [48, 49]. The criterion for the efficiency of sampling is the D-efficiency (see next footnote).

6 The D-efficiency is a measure of the orthogonality of the constellations of the vignette universe depicted in the sample, i.e. the full factorial. It takes on values between 0 and 100, where 100 represents complete orthogonality.

7 In the case of more levels, we would lose in efficiency considerably. If we increased the levels for the variables “working hours”, “autonomy”, “time for care recipient / patient”, “cordial team”, and “roster reliability” by one, we would only reach a D-efficiency of 65 with the original vignette sample of size 200.

8 Data access was provided within the context of a § 75 SGB X application.

9 The data are also the basis for a separate analysis on drivers of nurses’ job satisfaction, see Höld et al. [52].

10 A large part of the data cleansing consisted of checking whether respondents’ answers were consistent with the questionnaire filters.

11 Possible reasons for this are—besides differences in the willingness to participate in the interview—inter-alia (1) an erroneous record of the respondents’ characteristics within the data on which the sampling procedure is based (at least insofar as these are not relevant for the calculation of the social security contributions) and (2) changes in the variables between the time of the sampling procedure and the interview (in particular with regard to the status as former/active nurse or the care setting). To deal with these possible problems, we refer to the self-classification of the respondents in the survey to determine central individual information (former/active status, care setting). This is also sensible, as vignette ratings may be related to a person’s present status and setting rather than to possibly different information gathered at the time of sampling. Overall, it, therefore, does not seem sensible to reweight the sample because of the stated differences. It should be pointed out that the named differences may, if anything, affect the external validity of our results; the internal validity is not affected.

12 To check the sensitivity of our results, we also estimated RE ordered logit models, which account for the ordinal nature of our outcome variable. The results were nearly the same and are available from the authors upon request. As the proportional odds assumption on which the RE ordered logit model relies does not seem very realistic given that our outcome variable has so many levels, we chose to stick with the linear RE model.

13 To that end, we employed information for the year 2014 from the weakly anonymous Sample of Integrated Labour Market Biographies (SIAB) 1975—2014. The data were accessed on-site at the Research Data Centre (FDZ) of the Federal Employment Agency (BA) at the Institute for Employment Research (IAB) and/or via remote data access at the FDZ. Data documentation: Antoni et al. [56].

14 Nursing assistants who have undergone the respective vocational training.

15 Doiron et al. [6] evaluated the effect of a wage rise from the lowest to the highest level in their survey – an increase by over 55%. This seems to be an implausible wage increase for an otherwise similar position. A natural, data-driven approach would be the evaluation of changes by one standard deviation in the corresponding variable. The standard deviation of the log-wage variable in our vignette setup is 0.34. A wage change by 34 percent would yield the third highest effect on the attractiveness and the fourth highest effect on the acceptance measure. However, wage changes by over 30% still seem unrealistically high.

16 The change in $y$ due to a $\frac{\Delta wage}{wage}$ change in wages is given by $\Delta y={\beta }_{w}*\mathrm{log}(\frac{\Delta wage}{wage}+1)$.

17 The results would further be in line with what Doiron et al. [6] found in a comparable study for young Australian nurses, if Doiron et al. [6] had evaluated a more realistic change in wages than they did.

## Code availability

Analysis was conducted in Stata 16. Code can be made available to accredited researchers upon request.

## References

[1] Drennan VM, Ross F (2019). Global nurse shortages-the facts, the impact and action for change. Br. Med. Bull..

[2] Bundesagentur für Arbeit: Fachkräftesituation in Deutschland. https://statistik.arbeitsagentur.de/Navigation/Footer/Top-Produkte/Fachkraefteengpassanalyse-Nav.html, last checked March 2020 (2020). Accessed 10 Mar 2020

[3] Marć M, Bartosiewicz A, Burzyńska J, Chmiel Z, Januszewicz P (2019). A nursing shortage—a prospect of global and local policies. Int. Nurs. Rev..

[4] Buchan J, Twigg D, Dussault G, Duffield C, Stone PW (2015). Policies to sustain the nursing workforce: an international perspective. Int. Nurs. Rev..

[5] Andreassen L, Di Tommaso ML, Strøm S (2017). Nurses and physicians: a longitudinal analysis of mobility between jobs and labor supply. Empir. Econ..

[6] Doiron D, Hall J, Kenny P, Street DJ (2014). Job preferences of students and new graduates in nursing. Appl. Econ..

[7] Hanel B, Kalb G, Scott A (2014). Nurses' labour supply elasticities: the importance of accounting for extensive margins. J. Health Econ..

[8] Di Tommaso ML, Strøm S, Sæther EM (2009). Nurses wanted is the job too harsh or is the wage too low?. J. Health Econ..

[9] Shields MA (2004). Addressing nurse shortages: What can policy makers learn from the econometric evidence on nurse labour supply?. Econ. J..

[10] Antonazzo E, Scott A, Skatun D, Elliott RF (2003). The labour market for nursing: a review of the labour supply literature. Health Econ..

[11] Brewer CS, Kovner CT, Greene W, Tukov-Shuser M, Djukic M (2012). Predictors of actual turnover in a national sample of newly licensed registered nurses employed in hospitals. J. Adv. Nurs..

[12] Zeytinoglu IU, Denton M, Millen Plenderleith J (2011). Flexible employment and nurses’ intention to leave the profession: the role of support at work. Health Policy.

[13] Cunich M, Whelan S (2010). Nurse education and the retention of registered nurses in New South Wales. Econ. Rec..

[14] Kankaanranta T, Rissanen P (2008). Nurses' intentions to leave nursing in Finland. Eur. J. Health Econ..

[15] Frijters P, Shields MA, Price SW (2007). Investigating the quitting decision of nurses: panel data evidence from the British National Health Service. Health Econ..

[16] Holmås TH (2002). Keeping nurses at work. A duration analysis. Health Econ..

[17] Shields MA, Ward M (2001). Improving nurse retention in the National Health Service in England: the impact of job satisfaction on intentions to quit. J. Health Econ..

[18] Lu H, Zhao Y, While AE (2019). Job satisfaction among hospital nurses: a literature review. Int. J. Nurs. Stud..

[19] Lu H, Barriball KL, Zhang X, While AE (2012). Job satisfaction among hospital nurses revisited: a systematic review. Int. J. Nurs. Stud..

[20] Lu H, While AE, Barriball KL (2005). Job satisfaction among nurses. A literature review. Int. J. Nurs. Stud..

[21] Soekhai V, de Bekker-Grob EW, Ellis AR, Vass CM (2019). Discrete choice experiments in health economics: past present and future. Pharmacoeconomics.

[22] Fields BE, Bell JF, Bigbee JL, Thurston H, Spetz J (2018). Registered nurses' preferences for rural and urban jobs: a discrete choice experiment. Int. J. Nurs. Stud..

[23] Scott A, Witt J, Duffield C, Kalb G (2015). What do nurses and midwives value about their jobs? Results from a discrete choice experiment. J. Health Serv. Res. Policy.

[24] Auspurg K, Hinz T (2015). Factorial survey experiments. Quantitative applications in the social sciences.

[25] Bähr S, Abraham M (2016). The role of social capital in the job-related regional mobility decisions of unemployed individuals. Soc. Netw..

[26] Auspurg K, Gundert S (2015). Precarious employment and bargaining power: results of a factorial survey analysis. Z. Soziol..

[27] Abraham M, Auspurg K, Bähr S, Frodermann C, Gundert S, Hinz T (2013). Unemployment and willingness to accept job offers. Results of a factorial survey experiment. J. Labour Mark. Res..

[28] Trappmann M, Beste J, Bethmann A, Müller G (2013). The PASS panel survey after six waves. J. Labour Mark. Res..

[29] Song K, Scott A, Sivey P, Meng Q (2015). Improving Chinese primary care providers' recruitment and retention: a discrete choice experiment. Health Policy Plan..

[30] Saunders, K.M., Hagist, C., McGuire, A., Schlereth, C.: Nursing without caring? a discrete choice experiment about job characteristics of German surgical technologist trainees. WHU – Working Paper Series in Economics, 1–30 (2019)

[31] Bundesregierung, D.: Konzertierte Aktion Pflege Vereinbarungen der Arbeitsgruppen 1 bis 5, Berlin. https://www.bundesgesundheitsministerium.de/fileadmin/Dateien/3_Downloads/K/Konzertierte_Aktion_Pflege/0619_KAP_Vereinbarungstexte_AG_1-5.pdf (2019)

[32] Ariste R, Béjaoui A (2019). Impact of individual and institutional factors on wage rate for nurses in Canada: is there a monopsony market?. Int. Rev. Appl. Econ..

[33] Hirsch BT, Schumacher EJ (2005). Classic or new monopsony? Searching for evidence in nursing labor markets. J. Health Econ..

[34] Mangham LJ, Hanson K (2008). Employment preferences of public sector nurses in Malawi: results from a discrete choice experiment. Trop. Med. Int. Health.

[35] Huicho L, Miranda JJ, Diez-Canseco F, Lema C, Lescano AG, Lagarde M, Blaauw D (2012). Job preferences of nurses and midwives for taking up a rural job in Peru: a discrete choice experiment. PLoS ONE.

[36] Kunaviktikul W, Chitpakdee B, Srisuphan W, Bossert T (2015). Preferred choice of work setting among nurses in Thailand: a discrete choice experiment. Nurs. Health Sci..

[37] Kugler P (2021). The role of wage beliefs in the decision to become a nurse. Health Econ..

[38] Simon M, Müller BH, Hasselhorn HM (2010). Leaving the organization or the profession—a multilevel analysis of nurses' intentions. J. Adv. Nurs..

[39] Rockers PC, Jaskiewicz W, Kruk ME, Phathammavong O, Vangkonevilay P, Paphassarang C, Phachanh IT, Wurts L, Tulenko K (2013). Differences in preferences for rural job postings between nursing students and practicing nurses: evidence from a discrete choice experiment in Lao People's Democratic Republic. Hum. Resour. Health.

[40] Estryn-Behar M, van der Heijden BIJM, Fry C, Hasselhorn H-M (2010). Longitudinal analysis of personal and work-related factors associated with turnover among nurses. Nurs. Res..

[41] Beecroft PC, Dorey F, Wenten M (2008). Turnover intention in new graduate nurses: a multivariate analysis. J. Adv. Nurs..

[42] Bogai D (2017). Der Arbeitsmarkt für Pflegekräfte im Wohlfahrtsstaat.

[43] Bomball, J., Schwanke, A., Stöver, M., Schmitt, S.: Imagekampagne für Pflegeberufe auf der Grundlage empirisch gesicherter Daten. Einstellungen von Schüler/innen zur möglichen Ergreifung eines Pflegeberufs. IPP-Schriften, 05/2010. https://www.ipp.uni-bremen.de/uploads/IPPSchriften/ipp_schriften05.pdf (2010)

[44] Matthes, S.: Attraktivitätssteigerung durch Reform der Pflegeberufe. Hinweise aus einer Schülerbefragung. Fachbeiträge im Internet. https://www.bwp-zeitschrift.de/de/bwp.php/en/publication/show/8031 (2016)

[45] Simon M (2017). Das Gesundheitssystem in Deutschland.

[46] Harsch K, Verbeek H (2012). Der Mindestlohn in der Pflegebranche – Die Folgen eines Mindestlohns in einer Wachstumsbranche. J. Labour Mark. Res..

[47] Bogai, D., Carstensen, J., Seibert, H., Wiethölter, D., Hell, S., Ludewig, O.: Viel Varianz. Was man in den Pflegeberufen in Deutschland verdient. Der Beauftragte der Bundesregierung für die Belange der Patientinnen und Patienten sowie Bevollmächtigter für Pflege; Institut für Arbeitsmarkt und Berufsforschung, Berlin (2015)

[48] Johnson FR, Kanninen B, Bingham M, Özdemir S, Kanninen BJ (2006). Experimental design for stated-choice studies. Valuing environmental amenities using stated.

[49] Kuhfeld WF, Tobias RD, Garratt M (1994). Efficient experimental design with marketing research applications. J. Mark. Res..

[50] Sauer C, Auspurg K, Hinz T, Liebig S (2011). The application of factorial surveys in general population samples: the effects of respondent age and education on response times and response consistency. Surv. Res. Methods.

[51] Sauer C, Auspurg K, Hinz T, Liebig S, Schupp J (2014). Method effects in factorial surveys: an analysis of respondents' comments, interviewers' assessments, and response behavior. SSRN J..

[52] Höld J, Späth J, Kricheldorff C (2020). What makes them happy? Professional care-givers' job satisfaction. Z. Gerontol. Geriatr..

[53] Antoni, M., Schmucker, A., Seth, S., vom Berge, P.: Sample of integrated labour market biographies (SIAB) 1975–2017. FDZ-Datenreport, 02/2019 (en), Nürnberg (2019)

[54] Wooldridge JM (2010). Econometric analysis of cross section and panel data.

[55] Kliner, K., Rennert, D., Richter, M. (eds.): Gesundheit und Arbeit - Blickpunkt Gesundheitswesen. BKK Gesundheitsatlas 2017. MWV Medizinisch Wissenschaftliche Verlagsgesellschaft, Berlin (2017)

[56] Antoni, M., Ganzer, A., Vom Berge, P.: Sample of integrated labour market biographies (SIAB) 1975–2014. FDZ-Datenreport, 04/2016 (en), Nürnberg (2016)

[57] Dundler, A.: Befristete Beschäftigung. Methodische Hintergründe und Ergebnisse. Statistik der Bundesagentur für Arbeit, Grundlagen: Methodenbericht, Nürnberg (2018)

[58] Hackmann T (2010). Arbeitsmarkt Pflege: Bestimmung der künftigen Altenpflegekräfte unter Berücksichtigung der Verweildauer. Sozialer Fortschritt.

[59] Hackmann, T., Moog, S.: Pflege im Spannungsfeld von Angebot und Nachfrage. Diskussionsbeiträge, 33, Freiburg i. Br. http://hdl.handle.net/10419/38849 (2008)

[60] Long MC, Goldfarb MG, Goldfarb RS (2008). Explanations for persistent nursing shortages. Forum Health Econ. Policy.

[61] Bundestag, D.: Gesetz zur Reform der Pflegeberufe (Pflegeberufereformgesetz - PflBRefG). PflBRefG. In: Bundesgesetzblatt, vol. 2017, pp. 2581–2614 (2017)

